# Female athletes' knowledge of biopsychosocial puberty-related topics in sports. What is missing?

**DOI:** 10.3389/fspor.2025.1596418

**Published:** 2025-07-24

**Authors:** Felicia Radovan, Bård Erlend Solstad, Jørgen Bagger Kjær, Anna Melin, Ådne Ausland, Daniel Bjärsholm, Andreas Ivarsson, Monica Klungland Torstveit, Aron Laxdal, Sofia Ryman Augustsson, Susanne Linner

**Affiliations:** ^1^ Department of Sport Science, Linnaeus University, Kalmar/Växjö, Sweden; ^2^ Department of Sport Science and Physical Education, University of Agder, Kristiansand, Norway; ^3^ School of Health and Welfare, Halmstad University, Halmstad, Sweden

**Keywords:** interdisciplinary research, knowledge needs, perceived knowledge, puberty development, sports participation, subjective well-being

## Abstract

**Introduction:**

Puberty has been identified as one of the main contributing factors for girls dropping out of sports during adolescence. Knowledge and social support can, however, help athletes navigate the biopsychosocial (BPS) puberty-related changes associated with this period. Yet, research on female athletes' knowledge about BPS puberty-related topics is lacking. Therefore, this cross-sectional study aimed to examine female athletes' perceived knowledge and knowledge needs of BPS puberty-related topics during adolescence, explore interconnections across BPS domains, and investigate differences between athletes in team and individual sports.

**Method:**

A total of 1,323 Swedish and Norwegian female athletes (*M* age 18.7 ± 2.3 years, range 16–24) from ten sport disciplines [*n* = 657 (49.7%) team sport athletes; *n* = 656 (49.6%) individual sport athletes] completed an online survey in 2024 on perceived knowledge and knowledge needs regarding BPS puberty-related topics during adolescence.

**Results:**

Findings revealed low perceived knowledge and high knowledge needs among female athletes. A Mann–Whitney U test revealed higher perceived knowledge among team sport athletes regarding *individual differences, social cohesion, social comparison*, *acceptance within the group*, and *recovery*, compared to individual sport athletes. Additionally, team sport athletes reported higher knowledge needs regarding *social comparisons*, *sports nutrition* and *recovery*, while individual sport athletes reported higher knowledge needs regarding *changes in sex hormones*, *individual differences,* and *changes in body composition*. A network analysis identified clusters of *biological*, *psychosocial*, and *behavioral* topics in perceived knowledge and knowledge needs, indicating that knowledge is concentrated within domains.

**Discussion:**

The findings highlight gaps in knowledge among female athletes regarding BPS puberty-related topics across various sporting contexts. The results also underscore the urgent need for holistic and interdisciplinary educational programs addressing these knowledge gaps in the sporting context of female athletes.

## 1 Introduction

The decline in organized sports participation (hereby referred to as sports) during adolescence, with a particularly pronounced decline among girls in the ages of 12–16 years (1), is worrying. The changes that accompany puberty has been identified as one of the main contributing factors for girls dropping out of sports during adolescence (1–3). Indeed, puberty encompasses complex changes, including for example biological [e.g., the onset of menstruation and changes in body composition (2–5)], psychological [e.g., social comparisons and body image concerns (2, 4)], and social changes [e.g., peer influence and acceptance (4)]. While these changes occur separately, they are also interrelated—biopsychosocial (BPS)—adding to the complexity as athletes navigate in sports contexts during adolescence (6–8). Thus, the BPS model provides a useful framework as it integrates BPS components and how they relate to each other, thus allowing for a more holistic understanding (7, 9).

The BPS changes may pose challenges for girls in sports as they can influence both sports experiences and well-being (6, 8). Moreover, these challenges may differ across sports; in gymnastics, for example, puberty can conflict with sport-specific ideals such as the advantages of shorter stature (10, 11), whereas in sports like football and tennis, physical maturation may instead enhance performance through increased strength and power (11). Preparation, knowledge, and social support from parents and coaches can, however, make the changes associated with puberty more manageable while simultaneously enhance positive experiences and well-being (12–15). Conversely, the opposite is also possible—being uninformed, unprepared, and lacking social support—may contribute to more challenging experiences (12, 13). For example, feelings of unpreparedness of pubertal changes have been identified as a predictor of disordered eating among young women, underscoring one of the potential risks associated with insufficient knowledge and support during puberty (16). Hence, knowledge about puberty among girls and social support might be decisive for whether girls choose to continue or discontinue their sports participation during adolescence.

However, there appears to be a gap in research specifically exploring female athletes' understanding of BPS puberty within sports, as most studies have focused primarily on the biological aspects. For instance, in a systematic review of adolescent girls' knowledge and experiences of puberty and menstruation, the majority of studies (25/44) included knowledge of menstruation as an outcome, while only some (9/44) studies addressed knowledge of puberty (17). Based on this distribution, Coast et al. (17) suggested that there has been an elision of puberty with menstruation, which could explain the distribution and focus of previous research focus.

Another review has shown that both adolescent and adult female athletes generally lack knowledge of the menstrual cycle, and its potential impact on health and performance outcomes (18). Moreover, von Rosen et al. (19) found that only one-fourth of the female athletes (*M* age 24 years) perceived their own knowledge as “good” or “very good” regarding how sex hormone fluctuations during the menstrual cycle, and the use of hormonal contraceptives, affect the female athlete body. Most athletes also perceived their coaches' knowledge of the same topics as “poor” or “very poor” (19). Poor knowledge among coaches has also been highlighted in a qualitative study among adolescent female athletes (20). These athletes (*M* age 17.4 years) reported that support for menstruation in sports was insufficient, often due to a lack of education and stigma associated with it, including from coaches (20). Similarly, Larsen et al. (21) found that most female elite athletes aged 16 or older had in general limited knowledge about the menstrual cycle and oral contraceptives. Also, individual sport athletes had on average slightly higher knowledge than team sport athletes (21).

However, since puberty is inherently interdisciplinary and involves BPS changes that may influence sports experiences and well-being (6, 8), it is crucial to consider more than just the biological domain (6, 7). For instance, menstruation, a predetermined biological change during puberty, may influence the sports environment through psychological and social aspects, such as feelings of insecurity or anxiety about leakage (20). The BPS approach has previously been successfully applied in sport science research related to sport injuries (22, 23). However, to our knowledge, no research has previously examined female athletes' knowledge and knowledge needs about puberty across various BPS domains in sports.

Thus, this study aimed to (a) examine female athletes' perceived knowledge and knowledge needs regarding BPS puberty-related topics during adolescence and (b) explore interconnections across BPS domains. Additionally, this study sought to (c) investigate potential differences between athletes in team and individual sports.

## 2 Materials and methods

### 2.1 Study design and respondents

A cross-sectional online survey was conducted to collect data from Swedish and Norwegian female athletes (aged 16–24) in football, horseback riding, handball, swimming, gymnastics, floorball, athletics, cycling, and cross-country and alpine skiing. Sport disciplines were selected based on the highest participation rates among girls aged 13–16 years in Sweden (Skåne County) (24) and Norway (25). The 6-tiered Participant Classification Framework was applied to categorize the level of sport participation (26). To identify potential group differences (27), both team and individual sports were included. No comparative analyses between Swedish and Norwegian participants were conducted due to the uneven distribution of participants. Data were collected from February to June 2024.

### 2.2 Survey development

The survey focused on perceived knowledge and knowledge needs regarding BPS puberty-related topics during adolescence. In this study, *perceived knowledge* refers to the level of knowledge the respondents believed they had during adolescence, while *knowledge needs* refer to their perceived need for additional knowledge during adolescence. Both perceived knowledge and knowledge needs are related to *subjective well-being* in sports, referring to “a self-rated assessment of well-being in which an individual considers any events, circumstances, or experiences he or she is facing currently” (28, p. 2). This definition was further applied in sports, acknowledging that subjective well-being is a dynamic and multidimensional state, which can change depending on various circumstances (28, 29).

The survey was developed and reviewed by members of an interdisciplinary research group with expertise in survey construction. The process was guided by existing literature on female athletes BPS puberty development and sports participation to ensure that the items effectively captured the intended topics (30). The review enhanced content validity and ensured that the survey did not contain common errors, such as ambiguous or double-phased questions (31). The survey was deigned following Stockemer and Bordeleau's (31) guidelines, including ordering, groupings, and sections. Initially developed in Swedish, it also was translated into Norwegian by the project group using backward translation continuously during the process (32).

A pilot-test was conducted on a diverse subset of female athletes (*n* = 34) within the target age group to assess face validity (30). It also provided insights into readability, potential linguistic errors, and the time it took to complete the survey (31). The pilot test led to minor language adjustments and ensured that the translation was more likely to be correct (32).

The items in the survey were close-ended and included five items in the introduction, including (a) age, (b) age at entry into sports, (c) average training hours per week, (d) level of sport, and (e) type of sport. This was followed by 35 close-ended items divided into five main variables: *biological*, *psychosocial, behavioral, coaches* and *parents,* utilizing response options using a 7-point Likert-Scale.

The main variables *biological*, *psychosocial,* and *behavioral* included 24 items, addressing both perceived knowledge and knowledge needs of 12 BPS puberty-related topics: *changes in sex hormones* (e.g., breast development, pubertal fat mass, mood swings), *individual differences* (e.g., height growth and pubertal development), *the influence of biological puberty on sports development* (e.g., decreased motor control), *changes in body composition, menstruation* (e.g., bleeding frequency, menstrual pain, premenstrual symptoms such as rapid mood swings, fatigue, and low mood), *social cohesion* (e.g., sense of community, trust, and conversation climate), *social comparisons, acceptance within the group, feedback, unhealthy norms around body shape and weight, sports nutrition,* and *recovery* (e.g., sleep, rest). Perceived knowledge was assessed on a scale ranging from 1 (*very poor*) to 7 (*very good*) [e.g., “How would you rate your *perceived knowledge* during adolescence about how *changes in sex hormones* (e.g., breast development, pubertal fat mass, mood swings) could influence your well-being in sports?”]. Knowledge needs were assessed on a scale from 1 (*no degree*) to 7 (*high degree*) [e.g., “To what extent do you think *increased knowledge* about how *changes in sex hormones* (e.g., breast development, pubertal fat mass, mood swings) influence athletes could have enhanced your well-being in sports during adolescence?”].

The main variables *coaches* and *parents* consisted of five items each, addressing the following topics in a more general manner: *biological puberty development* (e.g., sex hormones, individual differences, impact on athletic development/performance, body composition and weight), *menstruation* (e.g., bleeding frequency, menstrual pain, premenstrual symptoms such as rapid mood swings, fatigue, and low mood), *psychosocial factors* (e.g., social cohesion, security within the group), *sports nutrition,* and *recovery* (e.g., sleep, rest). These items were assessed using a scale from 1 (*no degree*) to 7 (*high degree*) (e.g., “To what extent do you think increased knowledge about *biological puberty development* (e.g., sex hormones, individual differences, impact on athletic development/performance, body composition and weight) among your *coaches* could have enhanced your well-being in sports during adolescence?”).

Finally, the respondents were asked to assess the need among young female athletes' (13–16 years old) for increased knowledge regarding being a female athlete during puberty, rated on a scale from 1 (*no need*) to 7 (*very high need*).

### 2.3 Distribution of the online survey

Due to the EU's General Data Protection Regulation [SFS 2018:218; (33)], which prohibits associations from sharing personal information such as athletes' email addresses, the survey was primarily distributed through a two-step process across national districts in Sweden and Norway. Districts acted as gatekeepers, forwarding the survey to relevant sports clubs, which facilitated broader distribution and enhanced the potential sample representativeness.

### 2.4 Ethics approval statement

The study received ethical approval by the Ethics Review Authority in Sweden (Dnr. 2023-05264-01), and by the Norwegian Agency for Shared Services in Education and Research (ref: 930534), and the Research Ethics Committee at the Faculty of Health and Sport Science at the University of Agder (ref: RITM0225563) in Norway. The respondents gave their informed consent before responding the survey and could withdraw at any time.

### 2.5 Statistical analyses

Data were analyzed through both descriptive and analytical statistical methods in JASP (34). Descriptive statistics provided an overview of the data and response distribution, including central measures such as median and interquartile range for Likert scales.

To investigate potential differences between athletes in team and individual sports, the Mann–Whitney *U* test was used. A *p* < .05 was set to indicate statistically significant results. The Rank-Biseral correlation (rb), a non-parametric measure of effect-size based on correlation coefficient, was the calculated effect-size indicator for group differences (35). This effect-size indicator measures strength of associations in the *r*-family, varying from −1.0 to +1.0, with 0 indicating no effect. The strength of relationship can be interpreted through the following effect size stages: *small* (r = 0.10), *medium* (r = 0.24), and *large* (r = 0.37) (35).

To explore interconnections in knowledge across BPS domains, a Network Analysis was employed. This analysis combines multivariate statistics with network science, allowing for the exploration of associations within the data by identifying the system components—*nodes*—and the relationships between them—*edges* or *ties* (36, 37). This type of analysis provides insight into the structure and strength of these interconnections, highlighting which topics are central or peripheral within the network.

The analysis involved the generation of a network plot and a centrality plot. The network plot illustrates the relationships among nodes. In this study, nodes represented the 12 BPS puberty-related topics across all networks. The edges indicated the undirected relationships between them. Blue edges indicate positive connections, orange edges indicate negative connections, and thicker edges represent stronger relationships. The grouping of the nodes reflects their interconnectedness and *clustering* (36, 37).

The centrality measures *betweenness centrality*, *closeness centrality*, *strength centrality,* and *expected influence* were used to measure the centrality of the nodes in the networks (36). The node strength values are depicted on the *x*-axis of centrality plots, illustrating the node strengths of connections within the networks. Negative values indicate weaker or inhibitory connections, while positive values reflect stronger connections. Values near 0 suggest minimal importance in the network structure.

To illustrate network data, several figures were merged. Thus, the centrality plots display centrality measures for all respondents and differences between athletes in team and individual sports. Additionally, network plots for both athletes in team and individual sports were merged into one for clarity.

## 3 Results

The data collection yielded responses from 1,323 female athletes (*M* age 18.7 *±* 2.3 years), including 1,128 athletes from Norway and 195 from Sweden. All sport disciplines are shown in Table 1, with a total of 657 team sport athletes (49.7%) and 656 individual sport athletes (49.6%). Among these, 354 (26.8%) represented Tier 1, 309 (23.4%) Tier 2, 400 (30.2%) Tier 3, 166 (12.5%) Tier 4, and 94 (7.1%) Tier 5 (26). The mean age of entry into sport was 8.6 (*±*3.4) years and the mean training hours per week was 13.4 (*±*4.7) hours.

**Table 1:** Descriptives of all respondents in each sport discipline.

Sport	Frequency N (%)	*M* age	*M* age of entry into sport	*M* training hours per week	Type
Football	359 (27.1%)	19.2	8.6	11.5	Team
Handball	264 (20.0%)	19.1	8.7	11.6	Team
Cross-country skiing	239 (18.1%)	18.8	8.7	15.0	Individual
Gymnastics	103 (7.8%)	17.7	7.8	15.4	Individual
Athletics	90 (6.8%)	18.3	8.5	13.7	Individual
Swimming	83 (6.3%)	17.6	8.6	17.8	Individual
Alpine skiing	63 (4.8%)	18.2	10.2	15.1	Individual
Horseback riding	41 (3.1%)	18.8	8.4	13.7	Individual
Cycling	37 (2.8%)	19.2	8.9	18.6	Individual
Floorball	34 (2.6%)	18.2	7.6	9.0	Team
Others	10 (0.8%)	19.1	7.5	12.2	Not specified
Total	1,323 (100%)	18.7	8.6	13.4	

### 3.1 Perceived knowledge and knowledge needs

The results indicated that the respondents generally perceived their knowledge to be low (scores 2–3) across most of the included BPS puberty-related topics during adolescence (see Table 2). Scores were 2–3 for all topics, except for *feedback* among all respondents (score 4) and *social cohesion* among athletes in team sports (score 4; see Table 2). The results also revealed knowledge needs, as the respondents generally believed (scores of 5–6) that increased knowledge of all BPS puberty-related topics could have enhanced their subjective well-being in sports during adolescence (see Table 2).

**Table 2:** Differences in perceived knowledge and knowledge needs between female athletes in team and individual sports.

Topics	Knowledge	Group	Median	25th percentile	75th percentile	Mean Rank	U	*P*	Rank-Biseral Correlation (rb)
*Changes in sex hormones*	Perceived knowledge	Team	2.00	2.00	3.00	673.10	223315.00	0.237	0.036
		Individual	2.00	2.00	3.00	649.05			
	Knowledge needs	Team	5.00	4.00	6.00	612.78	183548.50	<.001	−0.148
		Individual	6.00	5.00	7.00	710.66			
*Individual differences*	Perceived knowledge	Team	3.00	2.00	4.00	684.40	231969.00	0.014	0.076
		Individual	2.00	2.00	3.00	634.13			
	Knowledge needs	Team	6.00	5.00	7.00	627.30	192882.00	<.001	−0.105
		Individual	6.00	5.00	7.00	696.62			
*The influence of biological puberty on sports development*	Perceived knowledge	Team	2.00	1.00	3.00	664.11	218015.00	0.703	0.012
		Individual	2.00	2.00	3.00	656.37			
	Knowledge needs	Team	6.00	5.00	7.00	644.61	204481.00	0.095	−0.051
		Individual	6.00	5.00	7.00	678.31			
*Changes in body composition*	Perceived knowledge	Team	2.00	2.00	3.00	662.96	216752.50	0.850	0.006
		Individual	2.50	2.00	3.00	659.10			
	Knowledge needs	Team	6.00	5.00	7.00	593.21	171065.00	<.001	−0.206
		Individual	6.00	6.00	7.00	729.48			
*Menstruation*	Perceived knowledge	Team	2.00	1.00	3.00	669.97	221798.00	0.345	0.029
		Individual	2.00	1.00	3.00	650.64			
	Knowledge needs	Team	6.00	5.00	7.00	654.04	210134.00	0.417	−0.025
		Individual	6.00	5.00	7.00	670.50			
*Social cohesion*	Perceived knowledge	Team	4.00	2.00	5.00	740.52	267911.50	<.001	0.243
		Individual	3.00	2.00	4.00	579.72			
	Knowledge needs	Team	6.00	4.00	7.00	642.46	203467.50	0.071	−0.056
		Individual	6.00	5.00	7.00	679.39			
*Social comparisons*	Perceived knowledge	Team	3.00	2.00	5.00	729.23	260443.50	<.001	0.209
		Individual	3.00	2.00	4.00	591.74			
	Knowledge needs	Team	6.00	5.00	7.00	701.31	242054.50	<.001	0.123
		Individual	5.00	4.00	6.25	620.08			
*Acceptance within the group*	Perceived knowledge	Team	3.00	2.00	5.00	695.88	239253.00	<.001	0.110
		Individual	3.00	2.00	4.00	623.12			
	Knowledge needs	Team	6.00	5.00	7.00	675.43	224888.50	0.159	0.044
		Individual	5.00	4.00	6.00	646.65			
*Feedback*	Perceived knowledge	Team	4.00	2.00	5.00	673.42	224708.00	0.173	0.043
		Individual	4.00	2.00	5.00	645.20			
	Knowledge needs	Team	6.00	5.00	7.00	670.87	221995.00	0.330	0.030
		Individual	6.00	5.00	7.00	650.90			
*Unhealthy norms around body shape and weight*	Perceived knowledge	Team	3.00	2.00	5.00	679.92	227450.00	0.076	0.055
		Individual	3.00	2.00	4.00	643.28			
	Knowledge needs	Team	6.00	4.00	7.00	656.84	213582.00	0.774	−0.009
		Individual	6.00	5.00	7.00	662.66			
*Sports nutrition*	Perceived knowledge	Team	3.00	2.00	5.00	671.89	222395.00	0.307	0.032
		Individual	3.00	2.00	5.00	650.68			
	Knowledge needs	Team	5.00	4.00	6.00	698.06	240718.50	<.001	0.117
		Individual	5.00	4.00	6.00	620.92			
*Recovery*	Perceived knowledge	Team	3.00	2.00	4.00	691.05	234881.50	0.004	0.090
		Individual	3.00	2.00	4.00	631.52			
	Knowledge needs	Team	5.00	4.00	6.00	696.87	240114.00	<.001	0.114
		Individual	5.00	4.00	6.00	621.53			

Median, 25th percentile, and 75th percentile reported for perceived knowledge and knowledge needs among female athletes in team (*n* = 657) and individual sports (*n* = 656). Perceived knowledge was measured on a 7-point Likert scale ranging from 1 (*very poor*) to 7 (*very good*). Knowledge needs were measured on a 7-point Likert scale ranging from 1 (*no* degree) to 7 (*high degree*). Mean rank represents the average ranking of groups in a Mann–Whitney *U* test. U = Mann–Whitney *U* value. *p* = probability value, where *p* < .05 indicates statistical significance. rb = rank-biserial correlation, which measures the strength and direction of the effect between groups.

Additionally, most respondents agreed on the need to increase knowledge among young female athletes (aged 13–16 years) about being a female athlete during puberty (scores of 6–7; see Supplementary Appendix S1).

#### 3.1.1 The athletes' coaches and parents

The respondents believed (scores 5–6) that increased knowledge about BPS puberty-related topics among their coaches and parents could have enhanced their subjective well-being in sports during adolescence (see Supplementary Appendix S2). The respondents rated *biological puberty development* and *menstruation* slightly higher in relation to coaches (score 6), while *sports nutrition* was slightly higher rated in relation to parents (score 6).

#### 3.1.2 Athletes in team and individual sports

Athletes in team sports reported significantly higher perceived knowledge during adolescence (highest MeanRanks) of *individual differences* (*p* < .014; rb 0.08), *social cohesion* (*p* < .001; rb 0.24), *social comparisons* (*p* < .001; rb 0.21), *acceptance within the group* (*p* < .001; rb 0.11) and *recovery* (*p* < .004; rb 0.09) compared to athletes in individual sports (see Table 2). The effect sizes (rb), presented in parentheses after each topic, ranged from 0.08–0.24.

Athletes in team sports reported significantly higher knowledge needs during adolescence (highest MeanRanks) of *social comparisons* (*p* < .001; rb 0.12), *sports nutrition* (*p* < .001; rb 0.12) and *recovery* (*p* < .001; rb 0.11) compared to athletes in individual sports (see Table 2). The effect sizes (rb) ranged from 0.11–0.12. Athletes in individual sports reported significantly higher knowledge needs during adolescence (highest MeanRanks) of *changes in sex hormones* (*p* < .001; rb −0.15), *individual differences* (*p* < .001; rb −0.11), and *changes in body composition* (*p* < .001; rb −0.21) compared to athletes in team sports (see Table 2). The effect sizes (rb) ranged from −0.21 to −0.11.

No significant differences were observed for the remaining topics of perceived knowledge or knowledge needs (*p* ≥ .071).

### 3.2 Interconnections in perceived knowledge

**Figure 1:**
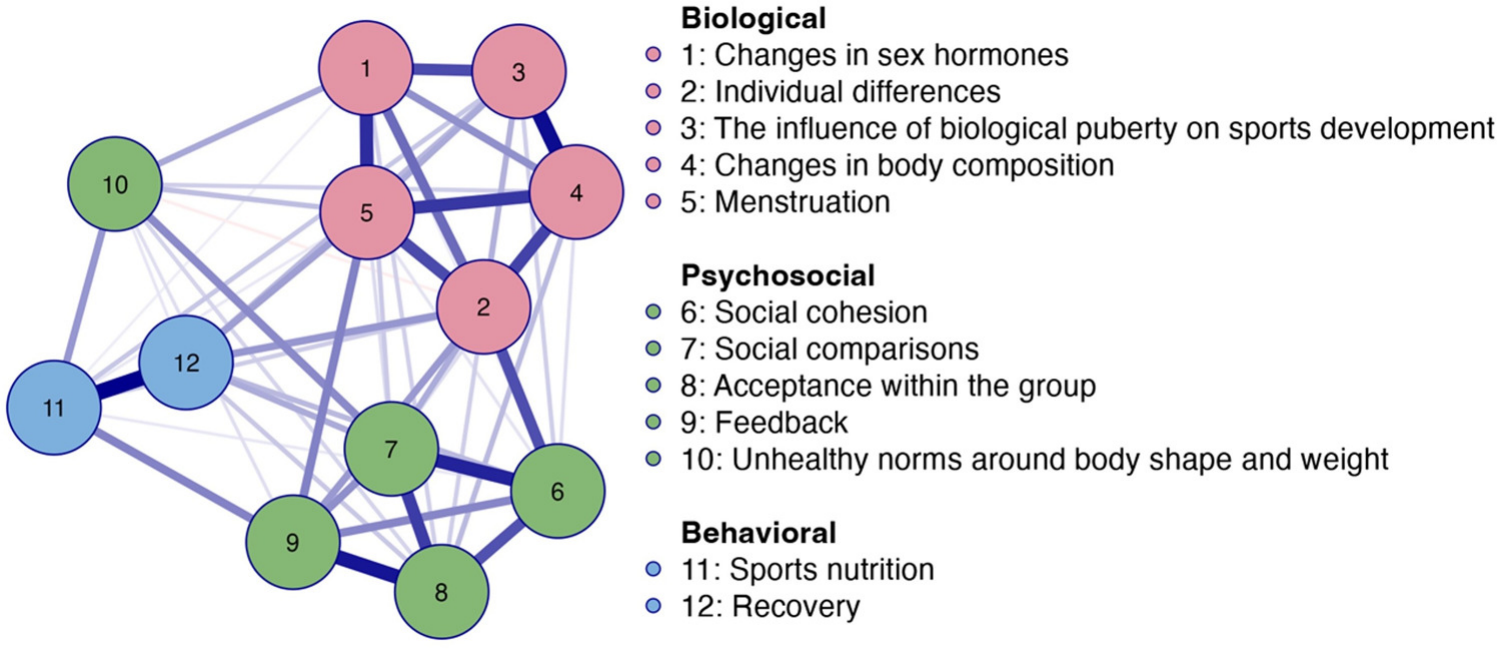
Network plot of perceived knowledge among all respondents (*n* = 1,323). Note: Blue edges represent positive connections, and orange edges represents negative connections. Thicker edges represent stronger relationships.

**Figure 2:**
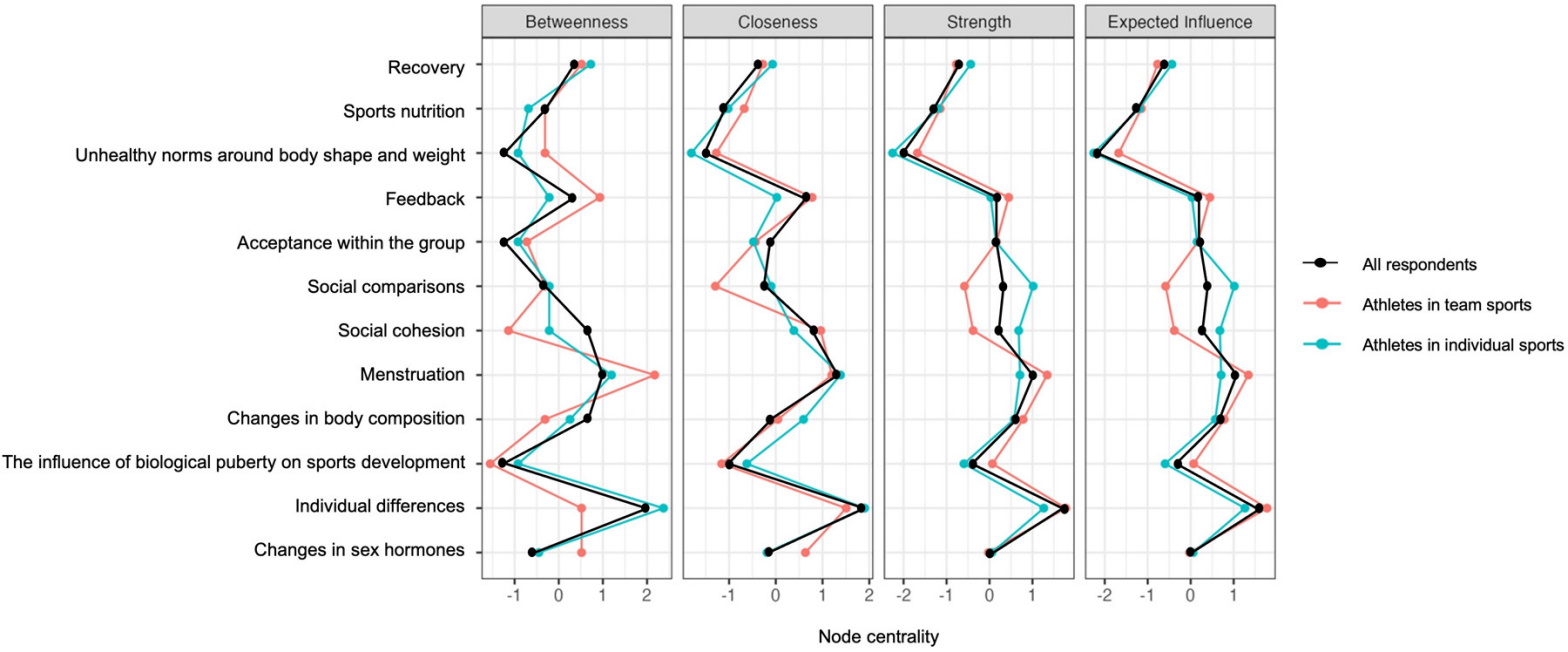
Centrality plot of perceived knowledge among all respondents (*n* = 1,323; black line), including a distinction between athletes in team (*n* = 657; orange line) and individual sports (*n* = 656; blue line). Note: Figure 2 merges network data from two previous figures covering all respondents as well as distinguishing between athletes in team and individual sports for enhanced clarity.

The network analysis of perceived knowledge among all respondents (see Figures 1, 2) identified 54 out of 66 non-zero edges, indicating that several topics were correlated. The overall network sparsity in Figure 1 was 0.182, corresponding to that 18.2% of the possible links were missing. The network analysis revealed a relatively dense network with distinct clusters, while some connections between topics remained absent.

According to the network plot (see Figure 1) and centrality plot (see Figure 2), *individual differences* appeared to be the most central node, characterized by strong connections and a high degree of influence on other nodes in the network. *Menstruation* and *social comparisons* were also central, with *menstruation* showing strong links to multiple nodes and high influence, whereas *social comparisons* had many connections but weaker associations. The least connected node in the network was *unhealthy norms related to body shape and weight*.

The network plot revealed three clusters of nodes, as illustrated in Figure 1. Due to strong interconnections, psychological and social topics merged into a single *psychosocial* cluster (9). This resulted in three clusters: a *biological* cluster (nodes 1–5), a *psychosocial* cluster (nodes 6–9, except for node 10 in perceived knowledge), and a *behavioral* cluster (nodes 11–12). These clusters indicated that perceived knowledge within each domain was more strongly related than across domains, suggesting that potential connections are currently not utilized. For instance, the node *acceptance within the group* appeared to be strongly linked to other psychosocial nodes (6, 7, 9) but was neither highly connected nor central within the overall network in Figure 1.

#### 3.2.1 Athletes in team and individual sports

**Figure 3:**
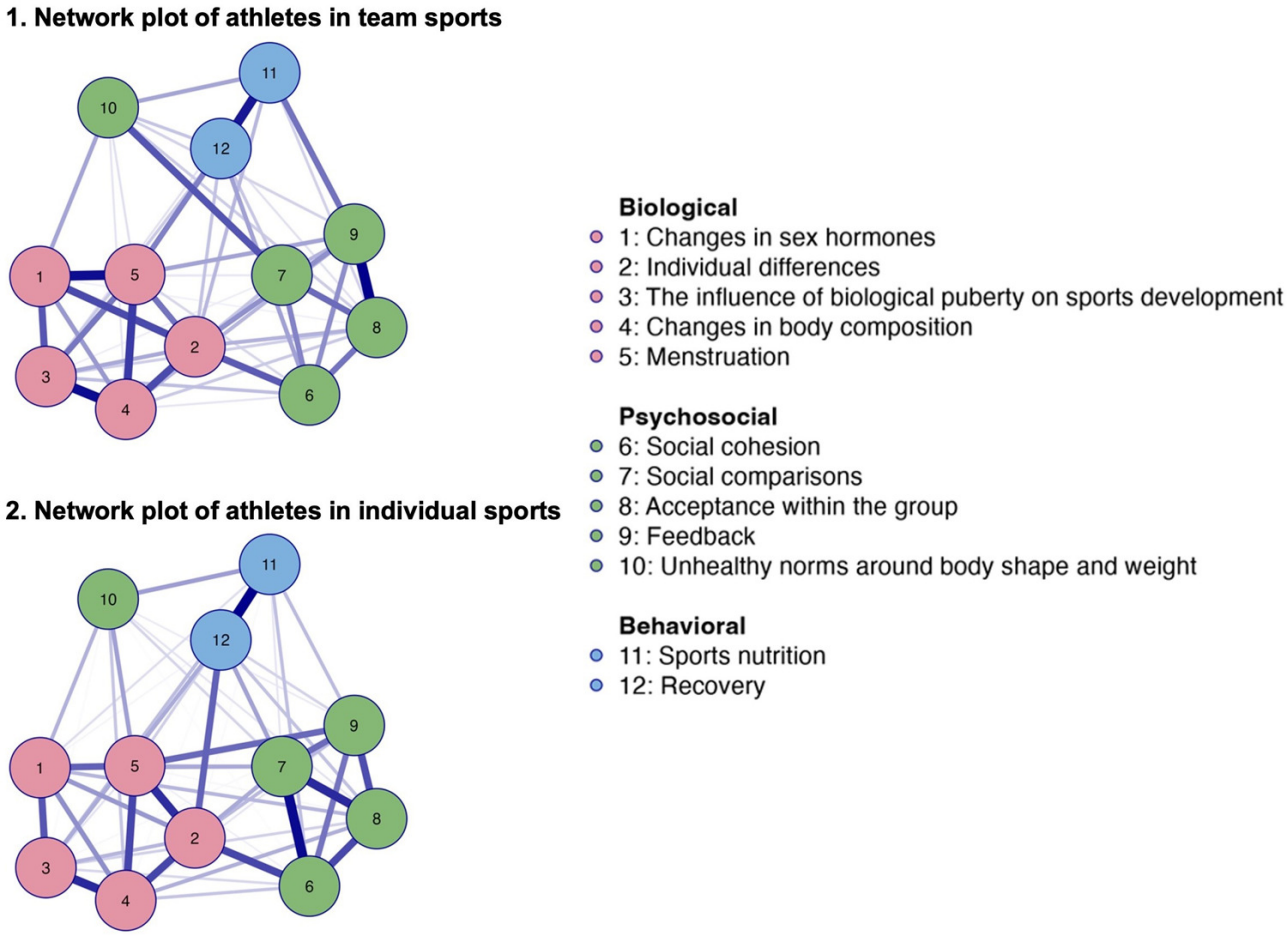
Network plots of perceived knowledge among athletes in team (*n* = 657) and individual sports (*n* = 656). Note: Blue edges represent positive connections, and orange edges represents negative connections. Thicker edges represent stronger relationships. Figure 3 merges network data from two previous figures covering athletes in both team and individual sports for enhanced clarity.

The network analysis of perceived knowledge among athletes in team sports, illustrated in Figure 3, plot 1*,* identified 53 out of 66 possible non-zero edges and an overall sparsity of 0.197. In contrast, the network of athletes in individual sports, illustrated in Figure 3, plot 2, identified of 58 out of 66 non-zero edges, and a lower overall sparsity of 0.121, compared to athletes in team sports.

*Individual differences, menstruation,* and *social comparisons* emerged as central nodes among both athletes in team and individual sports (see Figures 2, 3). The correlation between *unhealthy norms of body shape and weight* and *social comparisons* appeared to be stronger among athletes in team sports (see Figure 3, plot 1) compared to athletes in individual sports (see Figure 3, plot 2). The correlation between *individual differences* and *recovery* appeared to be stronger among athletes in individual sports (see Figure 3, plot 2) compared to athletes in team sports (see Figure 3, plot 1). *Social comparisons* seemed to be closer, stronger, and more influential in its connections in the network of athletes in individual sports (see Figure 2; Figure 3, plot 2).

The clusters were present within both networks, with the clusters of *biological* (nodes 1–5) and *behavioral* nodes (11 and 12) showing similar structures in both network plots (see Figure 3). The cluster of *psychosocial* nodes (6–9, with an exception for 10) appeared somewhat stronger among athletes in individual sports, as illustrated by the network plot in Figure 3, and further confirmed by the weight matrix (see Supplementary Materials S2, S3).

### 3.3 Interconnections in knowledge needs

**Figure 4:**
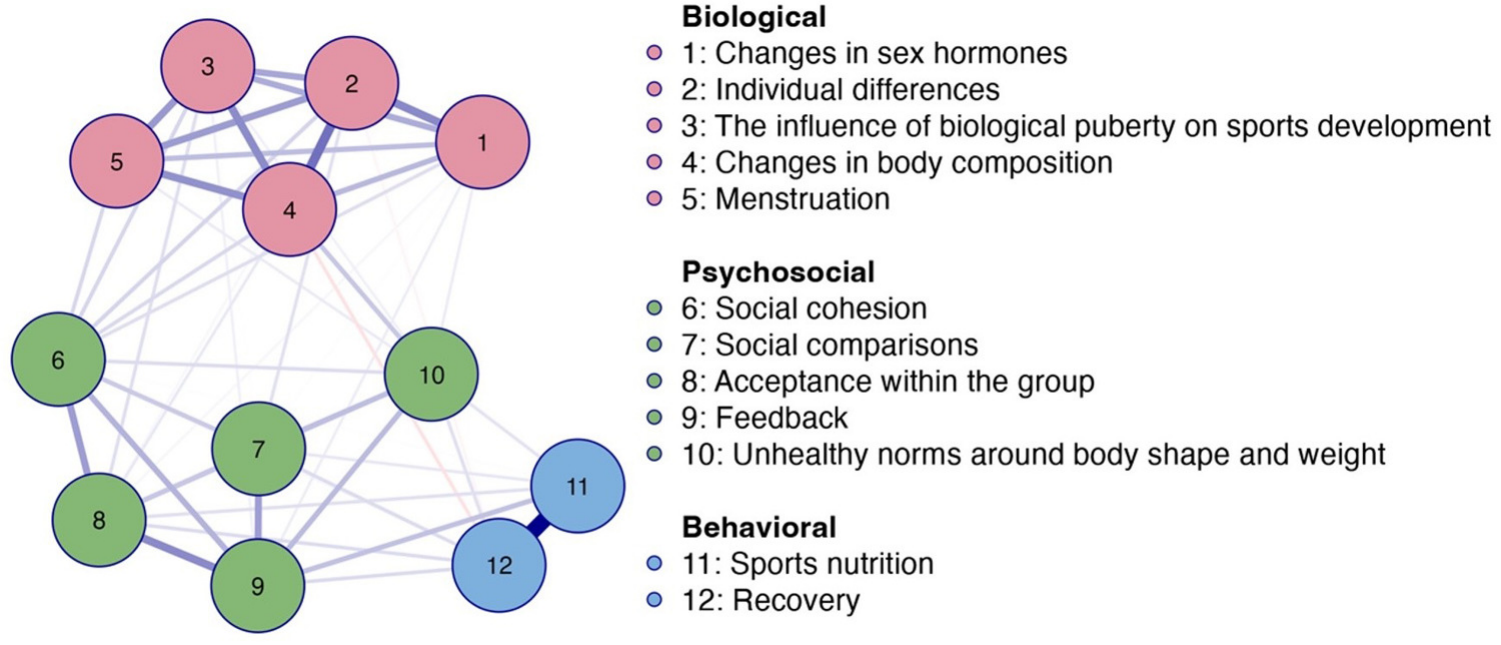
Network plot of knowledge needs among all respondents (*n* = 1,323). Note: Blue edges represent positive connections, and orange edges represents negative connections. Thicker edges represent stronger relationships.

**Figure 5:**
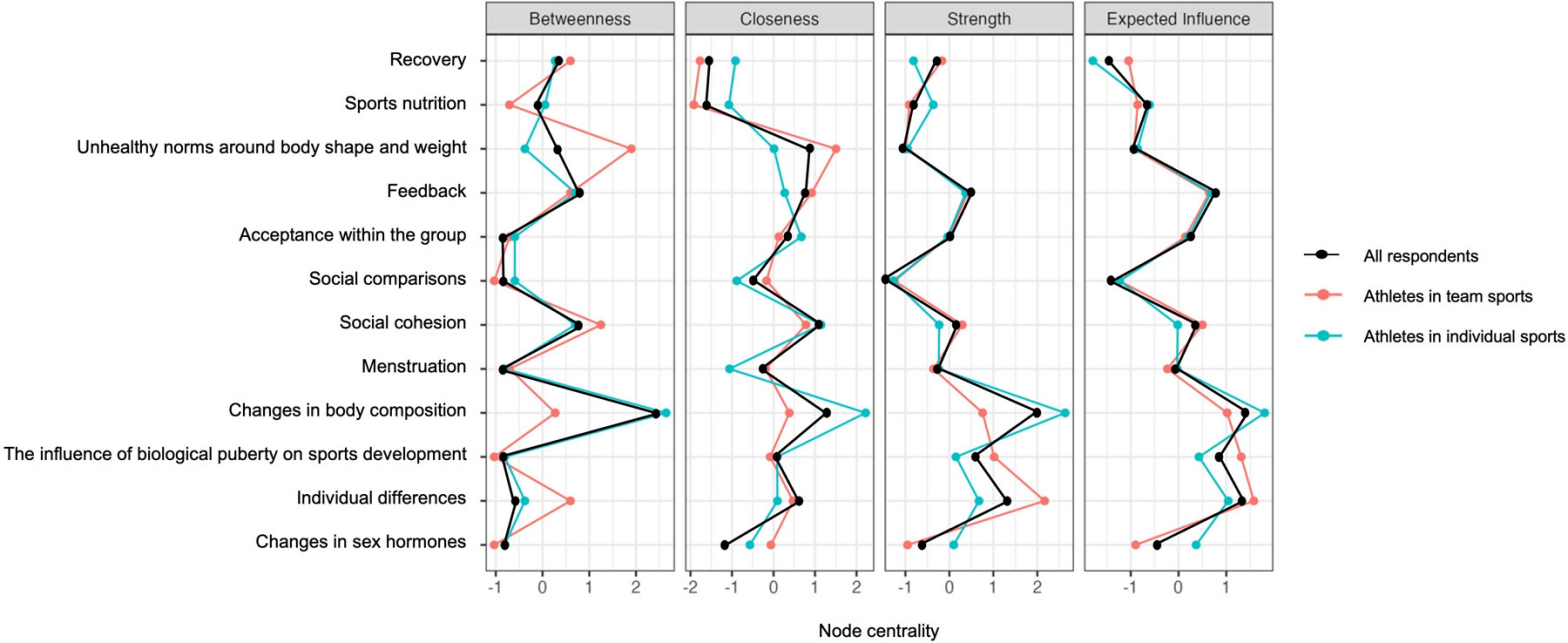
Centrality plot of knowledge needs among all respondents (*n* = 1,323; black line), including a distinction between athletes in team (*n* = 657; orange line) and individual sports (*n* = 656; blue line). Note: Figure 5 merges network data from two previous figures covering all respondents as well as distinguishing between athletes in team and individual sports for enhanced clarity.

The network analysis of knowledge needs among all respondents (see Figure 4 and Figure 5) identified 51 out of 66 possible non-zero edges and an overall sparsity of 0.227. Like previous network analyses (see Figures 1–3), this network (see Figure 4) appeared relatively dense although it still had some unconnected potential connections.

According to the network plot in Figure 4 and the centrality plot in Figure 5, *changes in body composition* emerged as the most central node in this network. Overall, the correlations between the nodes were weaker compared to previous network analyses. The clusters of *biological* (nodes 1–15), *psychosocial* (nodes 6–10), and *behavioral* nodes (11–12) persisted, with the node *unhealthy norms around body shape and weight* approaching the *psychosocial* cluster (see Figure 4).

#### 3.3.1 Athletes in team and individual sports

**Figure 6:**
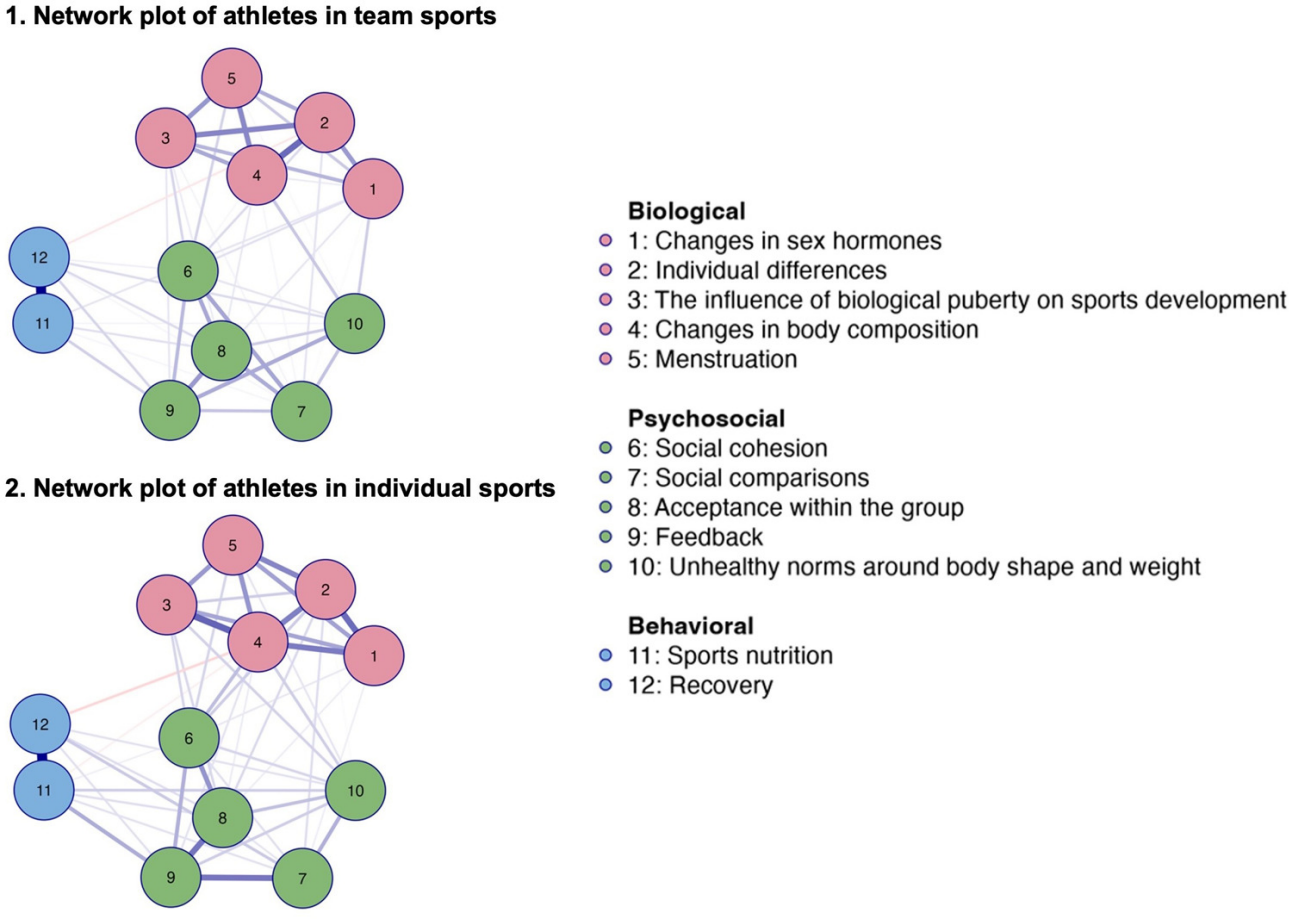
Network plots of knowledge needs among athletes in team (*n* = 657) and individual sports (*n* = 656). Blue edges represent positive connections, and orange edges represents negative connections. Thicker edges represent stronger relationships. Note: Figure 6 merges network data from two previous figures covering athletes in both team and individual sports for enhanced clarity.

The network analysis of knowledge needs among athletes in team and individual sports (see Figure 5 and Figure 6) identified 48 out of 66 non-zero edges, and an overall sparsity of 0.273 in both networks. The analysis showed sparser networks compared to previous networks (see Figures 1, 3) and similar patterns in knowledge needs between athletes in team and individual sports.

*Changes in body composition* appeared to be particularly central for athletes in individual sports, while *individual differences* were slightly more central for athletes in team sports (see Figures 5, 6).

The clusters were present in this network as well (see Figures 5, 6), and appeared to be slightly more isolated, although the correlations between the nodes were fewer and weaker (see Supplementary Materials S5, S6). As in the previous analysis of knowledge needs of all respondents (see Figures 4, 5), *unhealthy norms around body shape and weight* were included in the *psychosocial* cluster (nodes 6–10) (see Figure 6).

## 4 Discussion

The results revealed low perceived knowledge and high knowledge needs among female athletes regarding BPS puberty-related topics during adolescence. Clusters of *biological*, *psychosocial*, and *behavioral* topics indicated that knowledge is concentrated within BPS domains, rather than being evenly distributed across these domains. Differences were observed between athletes in team and individual sports in both perceived knowledge and knowledge needs.

The findings indicate that the respondents perceived their knowledge of BPS puberty-related topics to be low, reporting scores of 2–3 out of 7 in most topics. This aligns with previous research on female athletes' knowledge and awareness of puberty-related topics in domains, particularly in the biological domain (18–21). Moreover, the lack of perceived knowledge across all BPS domains align with the BPS model, suggesting that BPS domains are interconnected (7, 9, 38).

Team sport athletes perceived their knowledge to be significantly higher regarding *individual differences, social cohesion, social comparisons, acceptance within the group,* and *recovery,* compared to individual sport athletes. The effect sizes suggested small to medium differences between these groups, which may reflect the inherently social nature of team sports, where e.g., cooperation, competition for playing time, and social interactions shape athletes' sport experiences (39). In contrast to Larsen et al. (21), who found that individual sport athletes had, on average, slightly higher knowledge about the menstrual cycle and oral contraceptives, this study found no significant group differences in perceived knowledge of *menstruation*. Notably, despite the observed differences, both athletes in team and individual sports rated their overall knowledge as low, underscoring the need for increased knowledge among female athletes in general, as highlighted by previous research (15, 18–21).

The findings also revealed a substantial need for more knowledge, with respondents believing that increased knowledge of all BPS puberty-related topics could have enhanced their subjective well-being in sports during adolescence. The respondents not only recognized this need in themselves but also believed that increased knowledge among their coaches and parents could have enhanced their subjective well-being in sports during adolescence. These findings highlight the need for a more holistic understanding in the sporting context of female athletes (7, 9, 38) and the significance of the social network (12, 14). Specifically, knowledge needs related to biological topics were rated slightly higher in relation to coaches, while *sports nutrition* was rated slightly higher in relation to parents. This likely reflects their respective roles and tasks in relation to the child/athlete (40). Moreover, the need to increase knowledge among athletes, as well as their perceptions of the need for increased knowledge among coaches and parents, aligns with previous research (15, 18–21).

Furthermore, team sport athletes reported higher knowledge needs of *social comparisons, sports nutrition,* and *recovery* compared to individual sport athletes. The effect sizes suggested small differences between these groups, which could be attributed to the unique social dynamics of team sports, where athletes must navigate individual performance while simultaneously cooperating and competing with their teammates (39, 41). This complex task may in turn contribute to the feeling of increased knowledge needs related to performance and behavior-related topics as well as social comparisons with their competing teammates. In contrast, individual sport athletes reported greater knowledge needs of biological topics (*changes in sex hormones, individual differences,* and *changes in body composition*). The effect sizes suggested small to medium differences between the groups, potentially explained by the nature of individual sports, where athletes perform more individually and independently of their teammates (41). Thus, they are more dependent on their biological changes and independent of, for example, cooperation and the performance of their teammates.

The network analysis offered a nuanced understanding of the interconnectedness in knowledge — or lack thereof — between BPS domains (38). The clusters identified in perceived knowledge suggest that there is room for a more comprehensive understanding among female athletes. The 19% of missing connections among all respondents suggest a need for a more integrated understanding of BPS puberty-related topics among female athletes. Additionally, the higher proportion of missing connections among team sport athletes (20%) compared to individual sport athletes (12%) indicates a more cohesive understanding among individual sport athletes, particularly regarding psychosocial topics. This is interesting given that individual sport athletes in general rated their perceived knowledge lower than team sport athletes. One possible explanation is that individual sport athletes may engage in more self-reflection due to the individual nature of their sport, where they may attribute failures to internal factors (27, 41, 42). In contrast, team sport athletes may externalize challenges and attribute outcomes to group dynamics or their teammates (27, 41, 42). Another possible explanation may lie in the influence of group-based training environments in individual sports, despite the absence of direct competition among athletes—an aspect highlighted by Evans et al. (43). Thus, the more coherent understanding observed among athletes in individual sports could potentially be explained by a knowledge dissemination occurring even within individual sport groups and contexts (43).

The 23%–27% of missing connections and clusters in knowledge needs suggest that knowledge gaps within a topic are linked to related needs in the same domain, while certain connections between topics may also be unrecognized. This underscores the need for a more integrated understanding that strengthens insight into the interconnections between BPS puberty-related topics (6–8, 38).

The network analysis also identified key central nodes. *Individual differences, menstruation,* and *social comparisons* emerged as central nodes in perceived knowledge among the respondents, suggesting that these topics are more familiar. Additionally, it appeared that fewer respondents perceived themselves as having sufficient knowledge about how to manage *unhealthy norms around body shape and weight*, as this node was more distant in the networks. This may also suggest that *unhealthy norms around body shape and weight* are peripheral to the understanding of puberty or unevenly understood among participants. *Changes in body composition* emerged as the most central node in knowledge needs among all respondents and individual sport athletes, while *individual differences* emerged as the most central node among team sport athletes. This indicate that these topics are especially important to the respondents to gain more knowledge about. Furthermore, this may imply that it might be beneficial to target these topics in future interventions addressing knowledge needs as they may exert considerable influence on the other topics. Tailoring the intervention to the specific, identified knowledge needs within different sports contexts, such as team and individual sports, could enhance the overall understanding and subjective well-being of female athletes in their respective sports (44).

### 4.1 Strengths and limitations

The robust data material of >1,300 female athletes enabled a comprehensive understanding of Swedish and Norwegian female athletes' perceived knowledge and knowledge needs about BPS puberty-related topics in sports during adolescence. The interdisciplinary approach, including the BPS framework, and the network analysis enabled a nuanced perspective on knowledge of BPS puberty-related topics in sports (6–8, 38).

Nevertheless, the use of a non-validated questionnaire is acknowledged as a limitation and future research is encouraged to refine and validate the survey instrument. However, considering the absence of validated BPS questionnaires suitable for the intended study, this methodological choice was necessary to conduct the present study. The inability to contact respondents directly due to GDPR restrictions, necessitating the use of district gatekeepers for survey distribution, was also a limitation. While this approach ensured broad national coverage, it may have introduced non-response bias as certain districts were not willing to participate in this study (31). Additionally, self-selection bias may also have occurred due to some individuals being more likely to respond than others (31). Moreover, the retrospective design may have introduced response bias in the respondents recalls and self-reports (31). Lastly, the differences observed between athletes in team and individual sports may have been influenced by the specific sports included in this study (27). It is uncertain whether the results would have been the same if other sports, such as tennis or golf, had been considered instead of gymnastics and swimming—two leanness-focused sports (27).

### 4.2 Practical implications and future directions

The results of the present study highlight the urgent need of, and thus, a considerable opportunity for a more holistic and integrated understanding of BPS puberty in the sports contexts of female athletes. Differences in perceived knowledge and knowledge needs were identified between athletes in team and individual sports, suggesting potential advantages in tailoring future educational programs to their respective contexts. Moreover, the generally low perceived knowledge and the high knowledge needs observed among all respondents indicate that interventions are necessary for all athletes. Athletes recognized these knowledge needs not only within themselves but also in relation to their coaches and parents, suggesting that future educational programs should extend beyond athletes to include key stakeholders such as coaches and parents in addressing these knowledge gaps.

The findings of low perceived knowledge and high knowledge needs across BPS domains, and their clustering within these domains, suggest that it is insufficient to limit the scope of future educational programs to a single domain. Future educational programs should therefore be holistic and interdisciplinary in their scope when addressing these knowledge gaps in the sporting context of female athletes (6, 7, 38).

## 5 Conclusion

The present study revealed significant knowledge gaps, highlighting low perceived knowledge and high knowledge needs among female athletes regarding BPS puberty-related topics during adolescence. Team sport athletes perceived their knowledge to be slightly higher regarding *individual differences, social cohesion, social comparison*, *acceptance within the group*, and *recovery*, compared to individual sport athletes. Additionally, team sport athletes reported higher knowledge needs regarding *social comparisons*, *sports nutrition* and *recovery*, while individual sport athletes reported higher knowledge needs regarding *changes in sex hormones*, *individual differences,* and *changes in body composition*. The network analysis revealed clusters of *biological*, *psychosocial*, and *behavioral* topics in both perceived knowledge and knowledge needs, indicating that knowledge is concentrated within specific domains, rather than being evenly distributed across these domains. Consequently, our findings contribute to the field of sports science by highlighting gaps in knowledge regarding BPS puberty-related topics across various sporting contexts for female athletes. This underscores a significant opportunity to develop a more holistic and integrated understanding of puberty and sports participation in female athletes.

## Data Availability

The original contributions presented in the study are included in the article/Supplementary Material, further inquiries can be directed to the corresponding author.
