# Supplementary material for: Female athletes' knowledge of biopsychosocial puberty-related topics in sports. What is missing?
Source: Front Sports Act Living. 2025 Jul 24;7:1596418. doi: 10.3389/fspor.2025.1596418 (PMC12330288; doi:10.3389/fspor.2025.1596418)
Supplement: Supplementary file 1 [file Table1.docx]

Supplementary Material

# Appendices

Appendix 1

*All respondents (n=1323) perceived need to increase knowledge among young female athletes (aged 13-16).*

| **Perceived need** | **Frequency** | **Percent** | **Valid percent** | **Cumulative percent** |
| --- | --- | --- | --- | --- |
| 1 | 1 | 0.08 | 0.08 | 0.08 |
| 3 | 10 | 0.76 | 0.76 | 0.83 |
| 4 | 49 | 3.70 | 3.70 | 4.54 |
| 5 | 93 | 7.03 | 7.03 | 11.57 |
| 6 | 543 | 41.04 | 41.04 | 52.61 |
| 7 | 627 | 47.39 | 47.39 | 100.00 |
| Total | 1323 | 100.00 |  |  |

*Note:* Perceived need to increase knowledge was measured on a 7-point Likert scale ranging from 1 (no need); 7 (very high need).

Appendix 2

*Descriptives of all respondents perceived knowledge needs among their coaches and parents.*

| **Topics** | **Knowledge needs among their…** | **Median** | **25th percentile** | **75th percentile** |
| --- | --- | --- | --- | --- |
| *Biological puberty development* | Coach(es) | 6.00 | 5.00 | 7.00 |
|  | Parent(s) | 5.00 | 4.00 | 6.00 |
| *Menstruation* | Coach(es) | 6.00 | 5.00 | 7.00 |
|  | Parent(s) | 5.00 | 4.00 | 6.00 |
| *Psychosocial factors* | Coach(es) | 5.00 | 4.00 | 7.00 |
|  | Parent(s) | 5.00 | 4.00 | 6.00 |
| *Sports nutrition* | Coach(es) | 5.00 | 4.00 | 6.00 |
|  | Parent(s) | 6.00 | 5.00 | 7.00 |
| *Recovery* | Coach(es) | 5.00 | 4.00 | 7.00 |
|  | Parent(s) | 5.00 | 4.00 | 6.00 |

*Note:* Median, 25th percentile, and 75th percentile reported for all respondents perceived knowledge needs among their coaches (n=1323) and parents (n=1323). Perceived knowledge needs were measured on a 7-point Likert scale ranging from 1 (no degree) to 7 (high degree).

# Supplementary Data

**Supplementary material 1**

*Weight matrix from the network analysis of perceived knowledge among all respondents (n=1323).*

| **Topics ^a^** | **1** | **2** | **3** | **4** | **5** | **6** | **7** | **8** | **9** | **10** | **11** | **12** |
| --- | --- | --- | --- | --- | --- | --- | --- | --- | --- | --- | --- | --- |
| **1** | 0.000 | 0.144 | 0.174 | 0.107 | 0.205 | 0.020 | 0.052 | 0.046 | 0.000 | 0.083 | 0.017 | 0.000 |
| **2** | 0.144 | 0.000 | 0.076 | 0.183 | 0.176 | 0.171 | 0.080 | 0.037 | 0.098 | -0.018 | 0.049 | 0.102 |
| **3** | 0.174 | 0.076 | 0.000 | 0.236 | 0.081 | 0.047 | 0.000 | 0.047 | 0.000 | 0.000 | 0.054 | 0.068 |
| **4** | 0.107 | 0.183 | 0.236 | 0.000 | 0.194 | 0.032 | 0.034 | 0.066 | 0.052 | 0.046 | 0.000 | 0.000 |
| **5** | 0.205 | 0.176 | 0.081 | 0.194 | 0.000 | 0.027 | 0.034 | 0.000 | 0.122 | 0.063 | 0.021 | 0.094 |
| **6** | 0.020 | 0.171 | 0.047 | 0.032 | 0.027 | 0.000 | 0.214 | 0.169 | 0.117 | 0.000 | 0.022 | 0.068 |
| **7** | 0.052 | 0.080 | 0.000 | 0.034 | 0.034 | 0.214 | 0.000 | 0.189 | 0.108 | 0.113 | 0.000 | 0.081 |
| **8** | 0.046 | 0.037 | 0.047 | 0.066 | 0.000 | 0.169 | 0.189 | 0.000 | 0.228 | 0.041 | 0.000 | 0.053 |
| **9** | 0.000 | 0.098 | 0.000 | 0.052 | 0.122 | 0.117 | 0.108 | 0.228 | 0.000 | 0.030 | 0.121 | 0.000 |
| **10** | 0.083 | -0.018 | 0.000 | 0.046 | 0.063 | 0.000 | 0.113 | 0.041 | 0.030 | 0.000 | 0.101 | 0.023 |
| **11** | 0.017 | 0.049 | 0.054 | 0.000 | 0.021 | 0.022 | 0.000 | 0.000 | 0.121 | 0.101 | 0.000 | 0.248 |
| **12** | 0.000 | 0.102 | 0.068 | 0.000 | 0.094 | 0.068 | 0.081 | 0.053 | 0.000 | 0.023 | 0.248 | 0.000 |

*Note:* Each value represents the strength of the edges ranging from -1 to 1.

***^a^ Topics:*** 1. Changes in sex hormones; 2. Individual differences; 3. The influence of biological puberty on sports development; 4. Changes in body composition; 5. Menstruation; 6. Social cohesion; 7. Social comparisons; 8. Acceptance within the group; 9. Feedback; 10. Unhealthy norms around body shape and weight; 11. Sports nutrition; 12. Recovery.

**Supplementary material 2**

*Weight matrix from the network analysis of perceived knowledge among athletes in team sports (n=657).*

| **Topics ^a^** | **1** | **2** | **3** | **4** | **5** | **6** | **7** | **8** | **9** | **10** | **11** | **12** |
| --- | --- | --- | --- | --- | --- | --- | --- | --- | --- | --- | --- | --- |
| **1** | 0.000 | 0.180 | 0.168 | 0.101 | 0.240 | 0.008 | 0.023 | 0.014 | 0.000 | 0.089 | 0.000 | 0.000 |
| **2** | 0.180 | 0.000 | 0.084 | 0.179 | 0.140 | 0.163 | 0.053 | 0.076 | 0.110 | 0.000 | 0.071 | 0.060 |
| **3** | 0.168 | 0.084 | 0.000 | 0.242 | 0.135 | 0.064 | 0.016 | 0.048 | 0.000 | 0.000 | 0.028 | 0.052 |
| **4** | 0.101 | 0.179 | 0.242 | 0.000 | 0.196 | 0.025 | 0.060 | 0.053 | 0.073 | 0.026 | 0.000 | 0.000 |
| **5** | 0.240 | 0.140 | 0.135 | 0.196 | 0.000 | 0.039 | 0.012 | 0.000 | 0.091 | 0.030 | 0.038 | 0.125 |
| **6** | 0.008 | 0.163 | 0.064 | 0.025 | 0.039 | 0.000 | 0.123 | 0.138 | 0.112 | 0.000 | 0.000 | 0.093 |
| **7** | 0.023 | 0.053 | 0.016 | 0.060 | 0.012 | 0.123 | 0.000 | 0.147 | 0.075 | 0.170 | 0.000 | 0.054 |
| **8** | 0.014 | 0.076 | 0.048 | 0.053 | 0.000 | 0.138 | 0.147 | 0.000 | 0.254 | 0.050 | 0.045 | 0.029 |
| **9** | 0.000 | 0.110 | 0.000 | 0.073 | 0.091 | 0.112 | 0.075 | 0.254 | 0.000 | 0.044 | 0.140 | 0.000 |
| **10** | 0.089 | 0.000 | 0.000 | 0.026 | 0.030 | 0.000 | 0.170 | 0.050 | 0.044 | 0.000 | 0.089 | 0.058 |
| **11** | 0.000 | 0.071 | 0.028 | 0.000 | 0.038 | 0.000 | 0.000 | 0.045 | 0.140 | 0.089 | 0.000 | 0.230 |
| **12** | 0.000 | 0.060 | 0.052 | 0.000 | 0.125 | 0.093 | 0.054 | 0.029 | 0.000 | 0.058 | 0.230 | 0.000 |

*Note:* Each value represents the strength of the edges ranging from -1 to 1.

***^a^ Topics:*** 1. Changes in sex hormones; 2. Individual differences; 3. The influence of biological puberty on sports development; 4. Changes in body composition; 5. Menstruation; 6. Social cohesion; 7. Social comparisons; 8. Acceptance within the group; 9. Feedback; 10. Unhealthy norms around body shape and weight; 11. Sports nutrition; 12. Recovery.

**Supplementary material 3**

*Weight matrix from the network analysis of perceived knowledge among athletes in individual sports (n=656).*

| **Topics ^a^** | **1** | **2** | **3** | **4** | **5** | **6** | **7** | **8** | **9** | **10** | **11** | **12** |
| --- | --- | --- | --- | --- | --- | --- | --- | --- | --- | --- | --- | --- |
| **1** | 0.000 | 0.103 | 0.170 | 0.112 | 0.158 | 0.032 | 0.085 | 0.073 | 0.000 | 0.078 | 0.032 | 0.010 |
| **2** | 0.103 | 0.000 | 0.074 | 0.185 | 0.210 | 0.185 | 0.085 | 0.000 | 0.069 | 0.000 | 0.006 | 0.157 |
| **3** | 0.170 | 0.074 | 0.000 | 0.214 | 0.027 | 0.039 | 0.000 | 0.046 | 0.007 | 0.005 | 0.065 | 0.083 |
| **4** | 0.112 | 0.185 | 0.214 | 0.000 | 0.186 | 0.059 | 0.025 | 0.079 | 0.025 | 0.058 | 0.000 | 0.005 |
| **5** | 0.158 | 0.210 | 0.027 | 0.186 | 0.000 | 0.015 | 0.066 | 0.008 | 0.150 | 0.094 | 0.009 | 0.049 |
| **6** | 0.032 | 0.185 | 0.039 | 0.059 | 0.015 | 0.000 | 0.246 | 0.183 | 0.139 | 0.004 | 0.063 | 0.000 |
| **7** | 0.085 | 0.085 | 0.000 | 0.025 | 0.066 | 0.246 | 0.000 | 0.214 | 0.147 | 0.043 | 0.024 | 0.093 |
| **8** | 0.073 | 0.000 | 0.046 | 0.079 | 0.008 | 0.183 | 0.214 | 0.000 | 0.178 | 0.026 | 0.000 | 0.062 |
| **9** | 0.000 | 0.069 | 0.007 | 0.025 | 0.150 | 0.139 | 0.147 | 0.178 | 0.000 | 0.020 | 0.068 | 0.044 |
| **10** | 0.078 | 0.000 | 0.005 | 0.058 | 0.094 | 0.004 | 0.043 | 0.026 | 0.020 | 0.000 | 0.097 | 0.000 |
| **11** | 0.032 | 0.006 | 0.065 | 0.000 | 0.009 | 0.063 | 0.024 | 0.000 | 0.068 | 0.097 | 0.000 | 0.256 |
| **12** | 0.010 | 0.157 | 0.083 | 0.005 | 0.049 | 0.000 | 0.093 | 0.062 | 0.044 | 0.000 | 0.256 | 0.000 |

*Note:* Each value represents the strength of the edges ranging from -1 to 1.

***^a^ Topics:*** 1. Changes in sex hormones; 2. Individual differences; 3. The influence of biological puberty on sports development; 4. Changes in body composition; 5. Menstruation; 6. Social cohesion; 7. Social comparisons; 8. Acceptance within the group; 9. Feedback; 10. Unhealthy norms around body shape and weight; 11. Sports nutrition; 12. Recovery.

**Supplementary material 4**

*Weight matrix from the network analysis of knowledge needs among all respondents (n=1323).*

| **Topics ^a^** | **1** | **2** | **3** | **4** | **5** | **6** | **7** | **8** | **9** | **10** | **11** | **12** |
| --- | --- | --- | --- | --- | --- | --- | --- | --- | --- | --- | --- | --- |
| **1** | 0.000 | 0.204 | 0.142 | 0.121 | 0.113 | 0.056 | 0.003 | 0.014 | 0.029 | 0.032 | 0.000 | 0.000 |
| **2** | 0.204 | 0.000 | 0.151 | 0.247 | 0.171 | 0.073 | 0.050 | 0.012 | 0.000 | 0.000 | 0.000 | -0.018 |
| **3** | 0.142 | 0.151 | 0.000 | 0.198 | 0.177 | 0.070 | 0.003 | 0.055 | 0.025 | 0.024 | 0.000 | 0.000 |
| **4** | 0.121 | 0.247 | 0.198 | 0.000 | 0.191 | 0.064 | 0.000 | 0.036 | 0.000 | 0.095 | 0.000 | -0.050 |
| **5** | 0.113 | 0.171 | 0.177 | 0.191 | 0.000 | 0.068 | 0.000 | 0.000 | 0.000 | 0.034 | 0.000 | 0.000 |
| **6** | 0.056 | 0.073 | 0.070 | 0.064 | 0.068 | 0.000 | 0.091 | 0.169 | 0.131 | 0.060 | 0.003 | 0.011 |
| **7** | 0.003 | 0.050 | 0.003 | 0.000 | 0.000 | 0.091 | 0.000 | 0.094 | 0.166 | 0.112 | 0.040 | 0.058 |
| **8** | 0.014 | 0.012 | 0.055 | 0.036 | 0.000 | 0.169 | 0.094 | 0.000 | 0.201 | 0.095 | 0.052 | 0.059 |
| **9** | 0.029 | 0.000 | 0.025 | 0.000 | 0.000 | 0.131 | 0.166 | 0.201 | 0.000 | 0.117 | 0.107 | 0.061 |
| **10** | 0.032 | 0.000 | 0.024 | 0.095 | 0.034 | 0.060 | 0.112 | 0.095 | 0.117 | 0.000 | 0.048 | 0.052 |
| **11** | 0.000 | 0.000 | 0.000 | 0.000 | 0.000 | 0.003 | 0.040 | 0.052 | 0.107 | 0.048 | 0.000 | 0.444 |
| **12** | 0.000 | -0.018 | 0.000 | -0.050 | 0.000 | 0.011 | 0.058 | 0.059 | 0.061 | 0.052 | 0.444 | 0.000 |

*Note:* Each value represents the strength of the edges ranging from -1 to 1.

***^a^ Topics:*** 1. Changes in sex hormones; 2. Individual differences; 3. The influence of biological puberty on sports development; 4. Changes in body composition; 5. Menstruation; 6. Social cohesion; 7. Social comparisons; 8. Acceptance within the group; 9. Feedback; 10. Unhealthy norms around body shape and weight; 11. Sports nutrition; 12. Recovery.

**Supplementary material 5**

*Weight matrix from the network analysis of knowledge needs among athletes in team sports (n=657).*

| **Topics ^a^** | **1** | **2** | **3** | **4** | **5** | **6** | **7** | **8** | **9** | **10** | **11** | **12** |
| --- | --- | --- | --- | --- | --- | --- | --- | --- | --- | --- | --- | --- |
| **1** | 0.000 | 0.185 | 0.135 | 0.033 | 0.097 | 0.055 | 0.007 | 0.000 | 0.043 | 0.088 | 0.044 | 0.000 |
| **2** | 0.185 | 0.000 | 0.224 | 0.274 | 0.148 | 0.065 | 0.049 | 0.000 | 0.000 | 0.000 | 0.000 | -0.044 |
| **3** | 0.135 | 0.224 | 0.000 | 0.158 | 0.190 | 0.075 | 0.020 | 0.034 | 0.043 | 0.000 | 0.000 | 0.000 |
| **4** | 0.033 | 0.274 | 0.158 | 0.000 | 0.217 | 0.012 | 0.017 | 0.039 | 0.000 | 0.103 | 0.000 | 0.000 |
| **5** | 0.097 | 0.148 | 0.190 | 0.217 | 0.000 | 0.080 | 0.000 | 0.000 | 0.000 | 0.012 | 0.000 | 0.000 |
| **6** | 0.055 | 0.065 | 0.075 | 0.012 | 0.080 | 0.000 | 0.162 | 0.159 | 0.125 | 0.047 | 0.000 | 0.027 |
| **7** | 0.007 | 0.049 | 0.020 | 0.017 | 0.000 | 0.162 | 0.000 | 0.152 | 0.109 | 0.111 | 0.016 | 0.016 |
| **8** | 0.000 | 0.000 | 0.034 | 0.039 | 0.000 | 0.159 | 0.152 | 0.000 | 0.186 | 0.090 | 0.054 | 0.064 |
| **9** | 0.043 | 0.000 | 0.043 | 0.000 | 0.000 | 0.125 | 0.109 | 0.186 | 0.000 | 0.154 | 0.085 | 0.076 |
| **10** | 0.088 | 0.000 | 0.000 | 0.103 | 0.012 | 0.047 | 0.111 | 0.090 | 0.154 | 0.000 | 0.018 | 0.062 |
| **11** | 0.044 | 0.000 | 0.000 | 0.000 | 0.000 | 0.000 | 0.016 | 0.054 | 0.085 | 0.018 | 0.000 | 0.473 |
| **12** | 0.000 | -0.044 | 0.000 | 0.000 | 0.000 | 0.027 | 0.016 | 0.064 | 0.076 | 0.062 | 0.473 | 0.000 |

*Note:* Each value represents the strength of the edges ranging from -1 to 1.

***^a^ Topics:*** 1. Changes in sex hormones; 2. Individual differences; 3. The influence of biological puberty on sports development; 4. Changes in body composition; 5. Menstruation; 6. Social cohesion; 7. Social comparisons; 8. Acceptance within the group; 9. Feedback; 10. Unhealthy norms around body shape and weight; 11. Sports nutrition; 12. Recovery.

**Supplementary material 6**

*Weight matrix from the network analysis of knowledge needs among athletes in individual sports (n=656).*

| **Topics ^a^** | **1** | **2** | **3** | **4** | **5** | **6** | **7** | **8** | **9** | **10** | **11** | **12** |
| --- | --- | --- | --- | --- | --- | --- | --- | --- | --- | --- | --- | --- |
| **1** | 0.000 | 0.226 | 0.137 | 0.203 | 0.143 | 0.033 | 0.025 | 0.027 | 0.000 | 0.000 | 0.000 | 0.000 |
| **2** | 0.226 | 0.000 | 0.076 | 0.195 | 0.186 | 0.076 | 0.049 | 0.056 | 0.000 | 0.006 | 0.000 | 0.000 |
| **3** | 0.137 | 0.076 | 0.000 | 0.240 | 0.156 | 0.063 | 0.000 | 0.059 | 0.000 | 0.068 | 0.000 | 0.000 |
| **4** | 0.203 | 0.195 | 0.240 | 0.000 | 0.163 | 0.099 | 0.000 | 0.027 | 0.040 | 0.075 | -0.024 | -0.062 |
| **5** | 0.143 | 0.186 | 0.156 | 0.163 | 0.000 | 0.063 | 0.000 | 0.000 | 0.000 | 0.037 | 0.000 | 0.000 |
| **6** | 0.033 | 0.076 | 0.063 | 0.099 | 0.063 | 0.000 | 0.046 | 0.164 | 0.121 | 0.063 | 0.023 | 0.000 |
| **7** | 0.025 | 0.049 | 0.000 | 0.000 | 0.000 | 0.046 | 0.000 | 0.037 | 0.211 | 0.120 | 0.043 | 0.081 |
| **8** | 0.027 | 0.056 | 0.059 | 0.027 | 0.000 | 0.164 | 0.037 | 0.000 | 0.213 | 0.096 | 0.049 | 0.049 |
| **9** | 0.000 | 0.000 | 0.000 | 0.040 | 0.000 | 0.121 | 0.211 | 0.213 | 0.000 | 0.075 | 0.126 | 0.044 |
| **10** | 0.000 | 0.006 | 0.068 | 0.075 | 0.037 | 0.063 | 0.120 | 0.096 | 0.075 | 0.000 | 0.074 | 0.043 |
| **11** | 0.000 | 0.000 | 0.000 | -0.024 | 0.000 | 0.023 | 0.043 | 0.049 | 0.126 | 0.074 | 0.000 | 0.394 |
| **12** | 0.000 | 0.000 | 0.000 | -0.062 | 0.000 | 0.000 | 0.081 | 0.049 | 0.044 | 0.043 | 0.394 | 0.000 |

*Note:* Each value represents the strength of the edges ranging from -1 to 1.

***^a^ Topics:*** 1. Changes in sex hormones; 2. Individual differences; 3. The influence of biological puberty on sports development; 4. Changes in body composition; 5. Menstruation; 6. Social cohesion; 7. Social comparisons; 8. Acceptance within the group; 9. Feedback; 10. Unhealthy norms around body shape and weight; 11. Sports nutrition; 12. Recovery.
